# Mastication, swallowing, and salivary flow in patients with head and neck cancer: objective tests versus patient-reported outcomes

**DOI:** 10.1007/s00520-021-06368-6

**Published:** 2021-06-25

**Authors:** Jorine A. Vermaire, Cornelis P. J. Raaijmakers, Irma M. Verdonck-de Leeuw, Femke Jansen, C. René Leemans, Chris H. J. Terhaard, Caroline M. Speksnijder

**Affiliations:** 1 Department of Radiation Oncology, Imaging Division, University Medical Center Utrecht, Utrecht University, Utrecht, The Netherlands; 2 Department of Otolaryngology-Head and Neck Surgery and Cancer Center Amsterdam, Amsterdam UMC, Vrije Universiteit Amsterdam, Amsterdam, The Netherlands; 3 Department of Clinical, Neuro- and Developmental Psychology, Amsterdam Public Health Research Institute, Vrije Universiteit Amsterdam, Amsterdam, The Netherlands; 4 Department of Oral and Maxillofacial Surgery and Special Dental Care, University Medical Center Utrecht, Utrecht University, Heidelberglaan 100, PO Box 85.500, 3508 GA Utrecht, The Netherlands; 5 Department of Head and Neck Surgical Oncology, University Medical Center Utrecht, Utrecht University, Utrecht, The Netherlands

**Keywords:** EORTC QLQ-H&N35, GRIX, Head and neck cancer, Mixing ability test, Objective versus subjective data, Salivary flow test, 100-mL water swallow test, SWAL-QoL-NL

## Abstract

**Purpose:**

Before and after treatment for head and neck cancer (HNC), many patients have problems with mastication, swallowing, and salivary flow. The aim of this study was to investigate the association between objective test outcomes of mastication, swallowing, and salivary flow versus patient-reported outcomes (PROs) measuring mastication-, swallowing-, and salivary flow–related quality of life.

**Methods:**

Data of the prospective cohort “Netherlands Quality of Life and Biomedical Cohort Study” was used as collected before treatment, and 3 and 6 months after treatment. Spearman’s rho was used to test the association between objective test outcomes of the mixing ability test (MAT) for masticatory performance, the water-swallowing test (WST) for swallowing performance, and the salivary flow test versus PROs (subscales of the EORTC QLQ-H&N35, Swallow Quality of Life questionnaire (SWAL-QoL-NL) and Groningen Radiation-Induced Xerostomia (GRIX)).

**Results:**

Data of 142 patients were used, and in total, 285 measurements were performed. No significant correlations were found between the MAT or WST and subscales of the EORTC QLQ-H&N35. Significant but weak correlations were found between the MAT or WST and 4 subscales of the SWAL-QoL-NL. Weak to moderate correlations were found between the salivary flow test and GRIX at 3 and 6 months after treatment, with the highest correlation between salivary flow and xerostomia during the day (Spearman’s rho =  − 0.441, *p* = 0.001).

**Conclusion:**

The association between objective test outcomes and PROs is weak, indicating that these outcome measures provide different information about masticatory performance, swallowing, and salivary flow in patients with HNC.

**Supplementary Information:**

The online version contains supplementary material available at 10.1007/s00520-021-06368-6.

## Introduction

Head and neck cancer (HNC) is the seventh most common cancer worldwide, most often caused by alcohol and tobacco use, or the human papilloma virus (HPV) [1]. Treatment options for HNC (e.g., oral, pharyngeal, or laryngeal cancer) include surgery, radiation therapy (RT), and chemoradiation therapy (CRT). After treatment, patients may suffer from tissue fibrosis, osteoradionecrosis, xerostomia, or dysphagia. Deterioration in oral functioning (such as mastication, swallowing, saliva production, taste, dental condition, and speech) can result in complications such as malnutrition, dehydration, aspiration, and subsequent pneumonia. Within the first year after radiotherapy, approximately half of the HNC survivors experience difficulties with oral functioning, and unmet survivorship needs are common [2]. HNC survivors may experience psychosocial problems such as social isolation and depression, which decreases a person’s quality of life (QoL) [3–5].

To determine oral functioning before and after treatment, objective and subjective measures can be used. Objective measurements are based on how well a person can perform a task, irrespective of what they experience while performing the task. They are based upon an accurate representation of the world, and are therefore unbiased because they record only what is observed, without adding or taking away from the observation [6, 7]. A person’s subjective evaluation depends on individual values and priorities, which may differ between persons and even within persons. This subjective evaluation, or patient-reported outcome measure (PRO), is based on what people actually experience, and is increasingly being integrated in routine clinical practice [8, 9]. It has shown to contribute to improved communication, patient satisfaction, earlier detection of problems and subsequently earlier referral, and more efficient use of health services [8]. In order to develop strategies to reduce side effects of oncological treatment, it is important to know the relation between the patients’ subjective evaluation of his/her oral functioning and the objective function of the various organs involved. In previous research, multiple studies looked at this relation between objective and subjective measurements, especially comparing swallowing outcomes [10–13]. However, many different measures have been used, and there is a lack of consensus about a preferred method to measure swallowing performance [10]. In addition, most studies focus only on one part of oral functioning, or at one point in time. Therefore, in this paper, objective measures and PROs are compared for three main oral functions (mastication, swallowing, and salivary flow), using the same methodology in a large group of patients at different time points.

To measure more aspects of oral functioning in time, and in particular masticatory performance, dysphagia, and xerostomia, different tests can be used. Objective masticatory performance can be measured with, for example, comminution methods, sieving and optical scanning methods, gummy jelly as test food, and mixing ability methods [14]. One method using the mixing ability method (the mixing ability test (MAT)) has proven to be highly reliable in patients with HNC [15]. Objective swallowing performance can be measured with, for example, fiberoptic endoscopic evaluation of swallowing (FEES), or in a non-invasive and fast manner with minimal equipment using a 100-mL water swallow test (WST) [16, 17]. Measures of objective salivary flow rate from parotid and submandibular glands have been used for years to determine the dose–response relationship between RT dose and degree of hyposalivation or sticky saliva [18].

Subjective oral functioning can be measured with several validated questionnaires [19]. The European Organization for Research and Treatment of Cancer Quality of Life Core Questionnaire, Head and Neck module (EORTC QLQ-H&N35), was especially developed to measure HNC-specific problems and addresses different items of oral functioning [20]. The Dutch version of the Swallow Quality of Life questionnaire (SWAL-QoL-NL) was developed to address swallowing-specific problems [21]. The Groningen Radiation-Induced Xerostomia (GRIX) questionnaire was developed to observe xerostomia and sticky saliva during day and night [22]. Before creating prediction models that show patients at risk for developing mastication-, dysphagia-, or xerostomia-related problems after treatment, it is important to get insight in the association between objective and subjective measures to get a total image of oral functioning. Therefore, the aim of this study was to determine the association between the MAT, WST, or salivary flow test and the EORTC QLQ-H&N35, SWAL-QoL-NL or GRIX, before treatment, and 3 and 6 months after treatment.

## Methods

Data of the prospective cohort study Netherlands Quality of Life and Biomedical Cohort (Net-Qubic) Study were used [23]. Patients were recruited between 2014 and 2018 and included when they were 18 years or older, diagnosed with oral, oropharyngeal, hypopharyngeal, laryngeal, or unknown primary HNC. Patients with recurrent or residual disease, with cognitive impairments, and having trouble understanding or reading the Dutch language were excluded. The study protocol was approved by the Medical Ethics Committee (NL45051.029.13). In the present study, the study population consisted of patients with data on MAT, WST, and salivary flow test. These tests were only performed in one single center (University Medical Center Utrecht (UMCU)). Sociodemographic and clinical data about age, sex, tumor stage, tumor location, and treatment were collected from medical records. All participants signed informed consent. Data from objective tests and subjective questionnaires were used as collected before primary treatment (baseline, M0), 3 months after treatment (M3), and 6 months after treatment (M6). Patients that did not perform both objective and subjective measures at one time point were excluded. A comparison between objective and subjective data was based on assumptions regarding best fit of subjective data to objective data.

### Mixing ability test

The MAT consists of two layers of wax, with the colors red and blue (Plasticine modelling wax, non-toxic DIN EN-71, art. nos. crimson 52,801 and blue 52,809, Stockmar, Kalten Kirchen, Germany) [24]. The total thickness is 3 mm, with a diameter of 30 mm. The outcome variable ranges between 5 and 30, where a lower score implies a better mixed tablet and better masticatory performance. A subject was asked to chew on this tablet 20 times in order to mix the two colors. The tablet is then flattened, pressed to a thickness of 2 mm, and scanned on both sides using a high-quality scanner (Epson® V750, Long Beach, CA, USA). The scanned images are then processed using Adobe Photoshop CS3 extended (Adobe, San Jose, CA, USA). The histograms of both sides of the flattened and scanned wax tablet are added to obtain red and blue intensity distributions. The spread of the color intensities is measured [24]. In previous research, this test has proven to be highly reliable in patients with HNC (ICC = 0.886) [15].

### 100-mL water swallow test

During the WST, a subject was asked to drink 100 mL of water as quickly as is comfortably possible. The time to swallow this 100 mL (in seconds) and the number of swallows were counted, both by the subject and the researcher. Timing started when the water touched the bottom lip, and stopped when the larynx came to rest after the last swallow [25]. Persons failed the test when they coughed or choked post swallow, had a wet voice quality post swallow, or were unable to drink the whole 100 mL [17]. When a person was unable to drink the 100 mL, the residual water was measured and noted. In a previous research, this test has proven to be highly reliable in patients with HNC (ICC = 0.923 for number of swallows, and ICC = 0.893 for duration) [26].

### Saliva collection

Salivary flow was collected simultaneously from the floor of mouth (mainly submandibular gland) using a pipette, and from the left and right parotid gland using Lashley cups, as first described in 1981 [18]. The cups were placed over the orifice of the Stenson’s duct. Stimulation of the glands was achieved by applying one drop of citric acid to the mobile part of the tongue every minute, and collection was carried out for 10 min. The volume of saliva was measured as collected in tubes by weight, assuming the density of saliva as 1 g/ml. The flow rate was expressed in milliliters per 10 min (ml/10 min) for both parotid glands and the submandibular gland. In the present study, we used the total amount of saliva by adding up the saliva of both parotid glands and the submandibular gland. No oral stimulus was permitted for at least 30 min before saliva collection, including the WST and MAT [27]. In previous research, this test scored an ICC of 0.66 and 0.63 for the left and right parotid flow glands, indicating moderate test–retest reliability [28].

#### EORTC QLQ-H&N35

The EORTC QLQ-H&N35 is an additional questionnaire to the EORTC QLQ-C30 (core instrument), and widely used to measure QoL in patients with HNC [20]. It consists of 7 subscales: pain in the mouth (4 items), problems with swallowing (4 items), senses (2 items), speech (3 items), social eating (4 items), social contact (5 items), sexuality (2 items), and 11 single items which address problems with teeth, opening mouth, dry mouth, sticky saliva, coughing, feeling ill, painkillers, nutritional supplements, feeding tube, weight loss, and weight gain [29]. The scores are transformed to a scale of 0 to 100, with a higher score on the symptom scales implying a higher level of symptoms or problems [20]. In the present study, we used the subscales “pain in mouth” and “social eating,” and the single items “teeth,” “opening mouth,” “weight loss,” and “weight gain” to explore the association between these PROs and the MAT. The subscales “pain in mouth” and “problems with swallowing,” and the single items “dry mouth,” “coughing,” and “feeding tube” were used to explore the association between these PROs and the WST. The single items “dry mouth” and “sticky saliva” were used to explore the association between these PROs and the salivary output. This questionnaire performs well on internal consistency and construct validity, and is able to differentiate between diverse groups of patients regarding treatment, tumor size, time elapsed since treatment, and age [30]. In patients with HNC, Cronbach’s α ranges from 0.75 to 0.93 for most scales, indicating satisfactory internal consistency [20, 31].

### SWAL-QoL-NL

The SWAL-QoL-NL consists of 39 items on 9 subscales: general burden, food selection, eating duration, eating desire, fear of eating, mental health, social functioning, and symptoms [21, 29]. After completing, a total SWAL-QoL-NL score could be calculated based on 23 items (item 1–9 and 12–25). The scores range from 0 to 100, with a higher score indicating more impairment [29]. In the present study, we used the subscales “food selection,” “eating duration,” “eating desire,” “fear of eating,” and the total score to explore the association between these PROs and the MAT. We used the subscales “general burden,” “symptoms,” and the total score the explore the association between these PROs and the WST. Cronbach’s α ranges from 0.79 to 0.95 in patients with oropharyngeal dysphagia, and intraclass correlations range from 0.59 to 0.91, indicating excellent scale reliability [32].

#### GRIX

The GRIX consists of 14 questions and four subscales: xerostomia during day and night, and sticky saliva during day and night [22]. The scores were transformed to a scale from 0 to 100, with a higher score indicating more problems regarding xerostomia or sticky saliva. Total xerostomia and sticky saliva were calculated by adding up the day and night scores to get a score from 0 to 200. In the present study, all subscales were used to explore the association between the PROs and salivary flow. Cronbach’s α of these scales ranges between 0.82 and 0.94, and test–retest reliability was between 0.63 and 0.67, indicating moderate correlations [22].

### Statistical analyses

Data were tested for normality using a Shapiro–Wilk test. The associations between the WST, MAT, and salivary flow versus PROs were tested using Spearman’s rank correlation coefficient. The spearman correlation coefficient was categorized as very weak (0.0 to 0.1), weak (0.1 to 0.39), moderate (0.4 to 0.69), strong (0.7 to 0.89), and very strong (0.9 to 1.0) [33]. Scatterplots were created to visualize the MAT, WST, and salivary flow outcomes that had the highest correlation with one of the PROs. All analyses were performed using Statistical Package for the Social Sciences (SPSS) version 25 (Chicago, IL). A Bonferroni correction was used to account for the number of tests performed, in order to avoid a type Ι error [34]. This correction was calculated by dividing the p-value by the number of tests performed. The corrected p-value was 0.05/12 = 0.004 for the MAT, 0.05/8 = 0.006 for the WST, and 0.05/8 = 0.006 for the salivary flow. A *p*-value ≤ 0.004 or 0.006 was considered statistically significant.

## Results

**Fig. 1:**
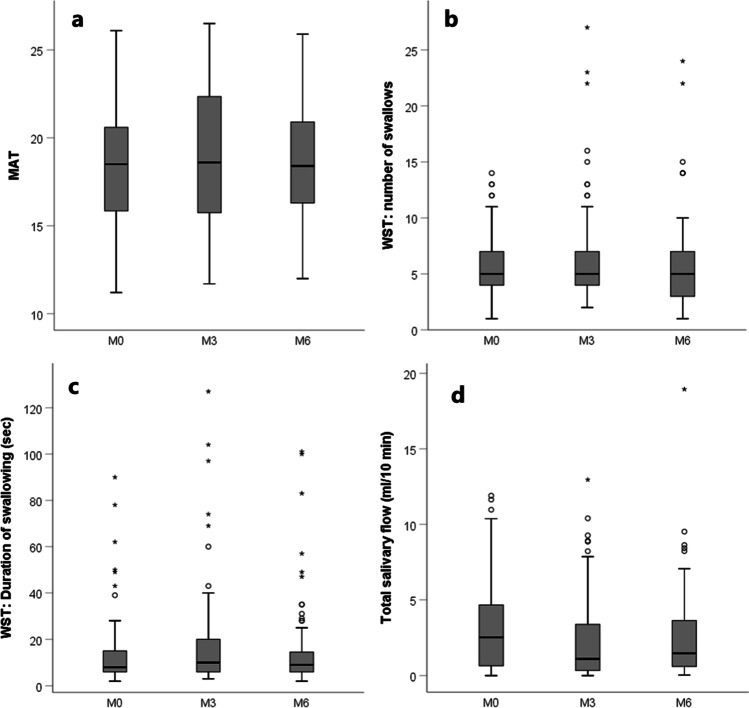
Boxplots displaying all objective measurements at M0, M3, and M6 for the MAT (**a**), number of swallows on the WST (**b**), duration of swallowing on the WST (**c**), and total salivary flow (**d**), respectively

The study cohort consisted of 142 patients out of the total Net-Qubic cohort in the UMCU (*N* = 154), of which 64 patients had repeated measurements for the MAT and WST at M0, M3, and M6. Twenty of these 142 patients had repeated measurements for the salivary flow measurements at M0, M3, and M6. Characteristics of patients can be found in Table 1. In total, 285 assessments for the MAT and WST were carried out: 101 at M0, 92 at M3, and 92 at M6. For the salivary flow measurements, 167 assessments were carried out: 45 at M0, 65 and M3, and 57 at M6. All data except the MAT at M0 and M6 were not normally distributed. Boxplots displaying the outcomes of the objective measurements can be found in Fig. 1. Regarding WST, there were missing data in 6 patients at M0 (5 patients because they were unable to drink the 100 mL, and 1 patient because of choking or coughing post swallow), in 12 patients at M3 (9 because they choked or coughed post swallow, and 3 because they were unable to drink the 100 mL), and in 9 patients at M6 (6 because they choked or coughed post swallow, and 3 because they were unable to drink the 100 mL).

**Table 1:** Characteristics of patients with HNC

	MAT and WST				Salivary flow test			
Characteristics	M0 (*N* = 101)	M3 (*N* = 92)	M6 (*N* = 92)	Repeated measurements M0, M3, and M6 (*N* = 64)	M0 (*N* = 45)	M3 (*N* = 65)	M6 (*N* = 57)	Repeated measurements M0, M3, and M6 (*N* = 20)
Age (median, IQR)	64.0 (15.5)	64.0 (13.8)	63.5 (13.8)	63.5 (14.0)	61.8 (16.0)	63.2 (15.0)	62.1 (13.5)	64.0 (11.0)
Sex								
Male	77 (76.2%)	72 (78.3%)	69 (75%)	49 (76.6%)	34 (75.6%)	55 (84.6%)	45 (78.9%)	15 (75.0%)
Female	24 (23.8%)	20 (21.7%)	23 (25%)	15 (23.4%)	11 (24.4%)	10 (15.4%)	12 (21.1%)	5 (25.0%)
Tumor site								
Oropharynx	36 (35.6%)	37 (40.2%)	34 (37.0%)	27 (42.2%)	14 (31.1%)	26 (40.0%)	23 (40.4%)	6 (30.0%)
Larynx	29 (28.7%)	24 (26.1%)	25 (27.2%)	16 (25.0%)	15 (33.3%)	18 (27.7%)	14 (24.6%)	6 (30.0%)
Oral cavity	27 (26.7%)	24 (26.1%)	26 (28.3%)	17 (26.6%)	11 (24.4%)	16 (24.6%)	16 (28.1%)	6 (30.0%)
Hypopharynx	3 (3.0%)	0 (0.0%)	3 (3.3%)	0 (0%)	1 (2.2%)	0 (0.0%)	1 (1.8%)	0 (0.0%)
Unknown primary	6 (5.9%)	7 (7.6%)	4 (4.3%)	4 (6.3%)	4 (8.9%)	5 (7.7%)	3 (5.3%)	2 (10.0%)
Tumor stage								
I	27 (26.7%)	22 (23.9%)	24 (26.1%)	16 (25.0%)	16 (35.6%)	16 (24.6%)	15 (26.3%)	6 (30.0%)
II	20 (19.8%)	17 (18.5%)	20 (21.7%)	15 (23.4%)	8 (17.8%)	15 (23.1%)	12 (21.1%)	3 (15.0%)
III	13 (12.9%)	13 (14.1%)	12 (13.0%)	6 (9.4%)	3 (6.7%)	7 (10.8%)	5 (8.8%)	2 (10.0%)
IV	41 (40.6%)	40 (43.5%)	36 (39.1%)	27 (42.2%)	18 (40.0%)	27 (41.5%)	25 (43.9%)	9 (45.0%)
Primary treatment								
RT	44 (43.6%)	42 (45.7%)	41 (44.6%)	30 (46.9%)	21 (46.7%)	31 (47.7%)	24 (42.1%)	8 (40.0%)
CRT	27 (26.7%)	25 (27.2%)	23 (25.0%)	18 (28.1%)	12 (26.7%)	17 (26.2%)	16 (28.1%)	7 (35.0%)
Surgery	20 (19.8%)	16 (17.4%)	19 (20.7%)	11 (17.2%)	8 (17.8%)	12 (18.5%)	10 (17.5%)	4 (20.0%)
Surgery with PO(C)RT	10 (9.9%)	9 (9.8%)	9 (9.8%)	5 (7.8%)	4 (8.9%)	5 (7.7%)	7 (12.3%)	1 (5.0%)

*M0* before treatment, *M3* 3 months after treatment, *M6* 6 months after treatment; *CRT* chemoradiation therapy, *IQR* interquartile range, *PO(C)RT* post-operative (chemo) radiation therapy, *RT* radiation therapy

### MAT versus PROs

**Fig. 2:**
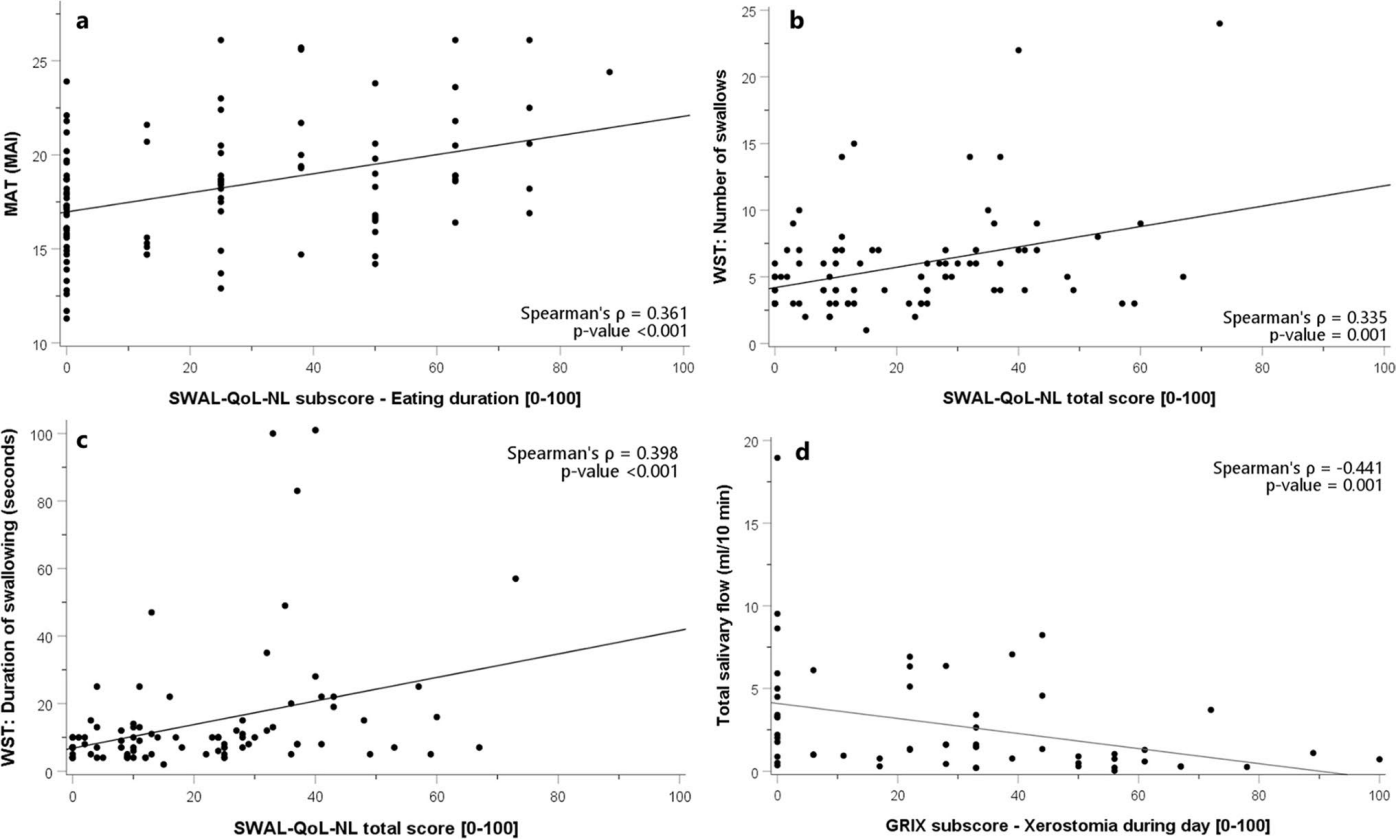
Scatterplots displaying the highest correlation for each objective measure; the eating duration on the SWAL-QoL-NL versus the MAT at M0 (**a**), the total score on the SWAL-QoL-NL questionnaire versus the number of swallows on the WST at M6 (**b**), and duration at M6 (**c**), and the xerostomia by day versus the total amount of saliva at M6 (**d**), respectively

The associations between MAT and subscales of the EORTC QLQ-H&N35 were not statistically significant at M0, M3, and M6 (Table 2). The association between the MAT and subscales of the SWAL-QoL-NL showed weak significant correlations at M0 for the items food selection (Spearman’s ρ = 0.347, *p* = 0.001), eating duration (Spearman’s ρ = 0.361, *p* < 0.001), fear of eating (Spearman’s ρ = 0.336, *p* = 0.001), and total SWAL-QoL-NL score (Spearman’s ρ = 0.310, *p* = 0.002). No significant correlations were found at M3 and M6. As an example, Fig. 2a displays the MAT versus the eating duration at M0. Correlations between the MAT and all items of the EORTC QLQ-H&N35, SWAL-QoL-NL, and GRIX are shown in Appendixes 1, 2, and 3, respectively.

**Table 2:** Spearman correlation coefficients of the MAT versus the EORTC QLQ-H&N35 and SWAL-QoL-NL

MAT	M0 (*N* = 101)		M3 (*N* = 92)		M6 (*N* = 92)	
	Spearman’s ρ	*p*-value	Spearman’s ρ	*p*-value	Spearman’s ρ	*p*-value
EORTC QLQ-H&N35						
Pain in mouth	0.082	0.417	0.066	0.541	− 0.014	0.896
Trouble with social eating	0.141	0.166	0.276	0.009*	0.201	0.054
Teeth	− 0.004	0.972	0.097	0.368	0.106	0.313
Opening mouth	0.026	0.803	0.211	0.048*	− 0.028	0.792
Feeding tube	− 0.102	0.319	0.146	0.173	− 0.040	0.703
Weight loss	− 0.119	0.247	− 0.004	0.968	− 0.009	0.936
Weight gain	0.058	0.571	0.004	0.970	0.086	0.412
SWAL-QoL-NL						
Food selection	0.347	0.001*†	0.227	0.034*	0.232	0.026*
Eating duration	0.361	< 0.001*†	0.154	0.152	0.185	0.078
Eating desire	0.167	0.105	0.014	0.900	0.211	0.043*
Fear of eating	0.336	0.001*†	0.172	0.108	0.163	0.119
Total score	0.310	0.002*†	0.165	0.124	0.222	0.033*

*M0* before treatment, *M3* 3 months after treatment, *M6* 6 months after treatment

^*^p ≤ 0.05

^†^*p* ≤ 0.004 (Bonferroni correction)

### WST versus PROs

The association between the PROs and the WST was calculated for both the number of swallows and the swallowing duration (Table 3). The association between the number of swallows and subscales of the EORTC QLQ-H&N35 showed no significant correlations at M0, M3, and M6. The association between the number of swallows and subscales of the SWAL-QoL-NL showed no significant correlations at M0 and M3. Weak significant correlations were found at M6 for the item total SWAL-QoL-NL score (Spearman’s ρ = 0.335, *p* = 0.001). The association between swallowing duration and subscales of the EORTC QLQ-H&N35 showed no significant correlations at M0, M3, and M6. The association between duration and subscales of the SWAL-QoL-NL showed no significant correlations at M0. At M3, weak significant correlations were found for the items food selection (Spearman’s ρ = 0.332, *p* = 0.001) and total SWAL-QoL-NL score (Spearman’s ρ = 0.353, *p* = 0.001). At M6, weak significant correlations were found for the item total SWAL-QoL-NL score (Spearman’s ρ = 0.398, *p* < 0.001). As example, Fig. 2b displays the total score on the SWAL-QoL-NL questionnaire versus the number of swallows of the WST, and Fig. 2c displays the total score on the SWAL-QoL-NL questionnaire versus the duration of the WST, both at M6. Correlations between the WST and all items of the EORTC QLQ-H&N35, SWAL-QoL-NL, and GRIX are shown in Appendixes 4, 5, and 6, respectively.

**Table 3:** Spearman correlation coefficients of the WST versus the EORTC QLQ-H&N35 and SWAL-QoL-NL

WST	Number of swallows						Duration					
	M0 (*N* = 101)		M3 (*N* = 92)		M6 (*N* = 92)		M0 (*N* = 101)		M3 (*N* = 92)		M6 (*N* = 92)	
	Spearman’s ρ	*p*-value	Spearman’s ρ	*p*-value	Spearman’s ρ	*p*-value	Spearman’s ρ	*p*-value	Spearman’s ρ	*p*-value	Spearman’s ρ	*p*-value
EORTC QLQ-H&N35												
Pain in mouth	0.088	0.378	0.175	0.094	− 0.009	0.936	0.125	0.205	0.253	0.015*	− 0.075	0.479
Swallowing	0.145	0.145	0.201	0.055	0.110	0.295	0.172	0.082	0.260	0.012*	0.176	0.094
Dry mouth	0.052	0.603	0.053	0.616	− 0.085	0.422	0.013	0.897	0.177	0.091	− 0.022	0.832
Coughing	− 0.018	0.859	0.035	0.741	− 0.056	0.595	0.090	0.364	0.092	0.385	0.018	0.866
SWAL-QoL-NL												
General burden	0.175	0.084	0.156	0.135	0.083	0.430	0.026	0.806	0.205	0.049*	0.162	0.123
Food selection	0.106	0.292	0.226	0.029*	0.244	0.020*	0.110	0.299	0.332	0.001*†	0.265	0.011*
Symptoms	0.248	0.013*	0.172	0.101	0.142	0.179	0.138	0.193	0.268	0.010*	0.255	0.015*
Total score	0.201	0.046*	0.238	0.023*	0.335	0.001*†	0.194	0.064	0.353	0.001*†	0.398	< 0.001*†

*M0*, before treatment, *M3*, 3 months after treatment, *M6*, 6 months after treatment

^*^p ≤ 0.05

^†^*p* ≤ 0.006 (Bonferroni correction)

### Salivary flow versus PROs

The association between total salivary flow and subscales of the EORTC QLQ-H&N35 and GRIX showed no significant differences at M0 (Table 4). At M3, weak significant differences were found for dry mouth (Spearman’s ρ =  − 0.339, *p* = 0.004) and sticky saliva (Spearman’s ρ =  − 0.321, *p* = 0.006) on the EORTC QLQ-H&N35, and for xerostomia during the day (Spearman’s ρ =  − 0.332, *p* = 0.006) on the GRIX questionnaire. At M6, weak significant differences were found for the item sticky saliva (Spearman’s ρ =  − 0.350, *p* = 0.006) on the EORTC QLQ-H&N35 and for the item sticky saliva during the day (Spearman’s ρ =  − 0.348, *p* = 0.006) on the GRIX questionnaire. A moderate correlation was found for the item xerostomia during the day (Spearman’s ρ =  − 0.441, p = 0.001) on the GRIX questionnaire. As an example, Fig. 2d displays xerostomia during the day versus total saliva at M6. Correlations between total salivary flow and all items of the EORTC QLQ-H&N35 and SWAL-QoL-NL can be found in Appendices 7 and 8, respectively.

**Table 4:** Spearman correlation coefficients of salivary flow versus the EORTC QLQ-H&N35 and GRIX

Total salivary flow	M0 (*N* = 45)		M3 (*N* = 65)		M6 (*N* = 57)	
	Spearman’s ρ	*p*-value	Spearman’s ρ	*p*-value	Spearman’s ρ	*p*-value
EORTC QLQ-H&N35						
Dry mouth	− 0.227	0.126	− 0.339	0.004*†	− 0.279	0.031*
Sticky saliva	− 0.217	0.143	− 0.321	0.006*†	− 0.350	0.006*†
GRIX						
Xerostomia during day	− 0.254	0.092	− 0.332	0.006*†	− 0.441	0.001*†
Xerostomia during night	− 0.147	0.329	− 0.225	0.063	− 0.099	0.450
Xerostomia total score	− 0.182	0.232	− 0.327	0.008*	− 0.320	0.015*
Sticky saliva during day	− 0.218	0.151	− 0.282	0.019*	− 0.348	0.006*†
Sticky saliva during night	− 0.299	0.044*	− 0.192	0.115	− 0.128	0.331
Sticking saliva total score	− 0.271	0.072	− 0.274	0.023*	− 0.256	0.048*

*M0* before treatment, *M3* 3 months after treatment, *M6* 6 months after treatment

^*^p ≤ 0.05

^†^p ≤ 0.006 (Bonferroni correction)

## Discussion

This study investigated associations between objective tests of mastication, swallowing, and salivary production and patient-reported outcomes. The associations between objective tests and PROs were weak (correlation below 0.40) for all items, except one: a moderate correlation between xerostomia during the day versus total salivary flow at M6 (Spearman’s ρ =  − 0.441, *p* = 0.001). In addition, none of the items on the EORTC QLQ-H&N35 questionnaire showed a significant correlation to the MAT or to the WST. Even when focusing on patients with the highest 10% scores on the MAT or WST, indicating worst masticatory or swallowing performance (see the top part in Fig. 2a–c), there was still a large variation of scores on the PROs taking up almost the entire scale. These findings indicate that the objective tests used in this research do not measure the same construct as the used PROs.

Our findings are in line with previous research in patients with HNC that showed that clinical measures and PROs generally correlate poorly [10, 12, 35]. For example, swallowing research showed weak to strong associations between the 100 mL WST and the MD Anderson Dysphagia Inventory (MDADI) questionnaire (a questionnaire similar to the SWAL-QoL) [10]. Research about salivary measurements found weak associations between salivary flow and xerostomia scores [36]. Other research stated that the EORTC quality of life questionnaire provided valuable data on subjective complaints, but that these complaints are not closely correlated with specific objective changes [37].

Previous research in patients with Parkinson’s disease and patients with schizophrenia showed that objective and subjective measures were not interchangeable, and each has a unique contribution to the problems assessed [38, 39]. Both objective and subjective measures may predict QoL in these patients [38]. Using PROs alone does not seem to measure function the same way functional tests do, and therefore should be combined with other data sources [9]. PROs are designed to assess how a patient evaluates his or her functioning rather than actual performance [40]. In addition, functional disorders measured via instrumental assessments by clinicians may not have a strong relationship with how patients perceive this disorder. Patients are more likely to rate their symptoms more severely than do clinicians, which can lead to an underestimation of side effects post-treatment [41]. Before selecting a measurement method, it is therefore important to identify the purpose of the measurement. For instance, when the effect of swallowing muscle sparing with RT is assessed, it is important to objectively test swallowing function. Whereas when the goal is to evaluate the effect of swallowing muscle sparing RT on perceived swallowing function of patients, it is important to use PROs. When only PROs are measured, it can be difficult to determine the cause of the reduced swallowing sensation. Individuals differ in what they find important, and expectations about one’s progress after treatment may change over time and in response to personal circumstances. Patients may develop a degree of adaptation over time, in which their PRO outcomes improve, but swallowing dysfunction stays the same or worsens [11]. Their subjective feeling of QoL also depends on satisfaction with, physical, material, emotional, and social well-being, and their development and activity. Objective observations record only what is observed; they are a representation of how something is [42]. The MAT, for example, reflects a complex process of oral muscle movements and coordination, which is difficult to answer with one single question in a questionnaire. Unfortunately, the loss of teeth, dental decay, and periodontal health were not assessed in this research, and may play an important role in oral functioning as well [37].

A limitation of this study was that the number of salivary flow measurements performed was smaller in comparison to that of the masticatory performance and swallowing measurements. Therefore, it is possible that these measurements are less reliable due to insufficient power. The salivary flow measurements were much more time-consuming, and difficulties occurred with the attachment of the Lashley cups. Therefore, it was chosen to combine the submandibular flow with the parotid flow and use the total flow to determine the associations between objective and subjective measures. Although these associations regarding xerostomia were higher in comparison to those of masticatory performance and swallowing measurements, a prerequisite should be that the objective test is easy and fast to perform, in order to be a valuable addition to PROs. A recommendation would therefore be to use an easy and fast test for measuring saliva flow, for example, by spitting saliva produced over a period of time in a plastic tube, with and without stimulation [43].

The SWAL-QoL-NL questionnaire is especially designed to detect swallowing problems. However, there is a close relationship between swallowing and mastication, as seen in multiple items such as “food selection,” “eating duration,” “eating desire,” and “fear of eating.” This relationship also comes across in many items of, e.g., the MDADI questionnaire [44]. When the focus is on swallowing-specific problems, it is therefore recommended to use swallowing-specific questions only, in combination with an objective swallowing test.

As shown in previous research, the reliability of the MAT, WST, EORTC QLQ-H&N35, and SWAL-QoL-NL was high, with a reliability of 0.886 for the MAT, and 0.893 and 0.923 for the WST. The reliability of the questionnaires was between 0.75 and 0.95, indicating that all measures are reliable to use in patients with HNC [15, 20, 26, 31, 32]. This study provides insight in the (weak) association between these objective and subjective measures. The results are important to take into account when developing prediction models to identify patients at risk for developing mastication, dysphagia, or xerostomia problems after treatment. Consistent with previous research, the results in this paper show that objective and subjective measures do not seem to measure the same construct, and therefore, separate prediction models with objective and subjective outcomes should be created, dependent on the aim of the model.

## Conclusion

This study showed significant but weak associations between objective tests of masticatory performance, swallowing, and salivary performance and patient-reported outcomes. It is therefore important to measure mastication-, dysphagia-, and xerostomia-related problems in patients with HNC both objectively and subjectively. This will acquire unique information and will help create the complete picture of patients’ perspective and functioning.

## Supplementary Information

Below is the link to the electronic supplementary material.

Supplementary file1 (PDF 615 KB)

## Data Availability

The collection and integration of large amounts of personal, biological, genetic, and diagnostic information precludes open access to the NET-QUBIC research data. In the section, data and sample dissemination (www.kubusproject.nl) describes how the data are made available for the research community.
