# Supplementary material for: Mastication, swallowing, and salivary flow in patients with head and neck cancer: objective tests versus patient-reported outcomes
Source: Support Care Cancer. 2021 Jun 25;29(12):7793–803. doi: 10.1007/s00520-021-06368-6 (PMC8550505; doi:10.1007/s00520-021-06368-6)
Supplement: Supplementary file 1 — Supplementary file1 (PDF 615 KB) [file 520_2021_6368_MOESM1_ESM.pdf]

Supplementary material

Mastication, swallowing and salivary flow in patients with head and neck cancer; objective tests versus patient reported outcomes

Supportive care in cancer

Jorine A. Vermaire, MSc, Cornelis P.J. Raaijmakers, MSc, PhD, Irma M. Verdonck-de Leeuw, MSc, PhD, Femke Jansen, PhD, C. René Leemans, MD, PhD, Chris H.J. Terhaard, MD, PhD, Caroline M. Speksnijder, PT, MSc, PhD[1]

1. *Department of Oral and Maxillofacial Surgery and Special Dental Care*

   University Medical Center Utrecht, G05.122

   P.O. Box 85.500

   3508 GA Utrecht

   The Netherlands

   e-mail: C.M.Speksnijder@umcutrecht.nl

Appendix 1. Spearman correlation coefficients of the MAT versus the EORTC QLQ-H&N35

**MAT**<br>**EORTC QLQ-H&N35**	***M0***<br>***(N=101)***		***M3***<br>***(N=92)***		***M6***<br>***(N=92)***	
	Spearman's ρ	*p-value*	*Spearman's ρ*	*p-value*	*Spearman's ρ*	*p-value*
Pain in mouth	0.082	0.417	0.066	0.541	-0.014	0.896
Swallowing	0.103	0.312	0.123	0.253	0.245	0.018*
Senses problems	0.120	0.238	0.198	0.065	0.101	0.337
Speech problems	0.009	0.929	0.200	0.062	0.156	0.135
Trouble with social eating	0.141	0.166	0.276	0.009*	0.201	0.054
Trouble with social contact	0.029	0.778	0.295	0.005*	0.209	0.045*
Less sexuality	-0.048	0.642	0.084	0.447	0.187	0.078
Teeth	-0.004	0.972	0.097	0.368	0.106	0.313
Opening mouth	0.026	0.803	0.211	0.048*	-0.028	0.792
Dry mouth	-0.035	0.734	-0.032	0.766	-0.122	0.243
Sticky saliva	-0.053	0.605	-0.001	0.995	-0.073	0.487
Coughing	0.102	0.317	0.186	0.083	0.143	0.171
Feeling ill	0.013	0.903	0.113	0.296	0.104	0.324
Pain killers	0.135	0.186	0.068	0.530	0.069	0.510
Nutritional supplements	0.163	0.109	0.214	0.045*	0.089	0.395
Feeding tube	-0.102	0.319	0.146	0.173	-0.040	0.703
Weight loss	-0.119	0.247	-0.004	0.968	-0.009	0.936
Weight gain	0.058	0.571	0.004	0.970	0.086	0.412

*:p≤0.05; M0: before treatment, M3: 3 months after treatment, M6: 6 months after treatment

Appendix 2. Spearman correlation coefficients of the MAT versus the SWAL-QoL-NL

**MAT**<br>**SWAL-QoL-NL**	***M0***<br>***(N=101)***		***M3***<br>***(N=92)***		***M6***<br>***(N=92)***	
	Spearman's ρ	*p-value*	*Spearman's* ρ	*p-value*	*Spearman's* ρ	*p-value*
General burden	0.264	0.010*	0.032	0.767	0.150	0.151
Food selection	0.347	0.001*	0.227	0.034*	0.232	0.026*
Eating duration	0.361	<0.001*	0.154	0.152	0.185	0.078
Eating desire	0.167	0.105	0.014	0.900	0.211	0.043*
Fear of eating	0.336	0.001*	0.172	0.108	0.163	0.119
Mental health	0.323	0.001*	0.112	0.299	0.204	0.051
Social functioning	0.247	0.016*	0.225	0.035*	0.202	0.052
Symptoms	0.385	<0.001*	0.239	0.025*	0.292	0.005*
**Total score**	0.310	0.002*	0.165	0.124	0.222	0.033*

*: $p \leq 0.05$; M0: before treatment, M3: 3 months after treatment, M6: 6 months after treatment

Appendix 3. Spearman correlation coefficients of the MAT versus the GRIX

**MAT**<br>**GRIX**	***M0***<br>***(N=93)***		***M3***<br>***(N=82)***		***M6***<br>***(N=89)***	
	Spearman's ρ	*p-value*	*Spearman's ρ*	*p-value*	*Spearman's ρ*	*p-value*
Xerostomia during day	0.008	0.936	-0.022	0.843	-0.065	0.548
Xerostomia during night	0.126	0.223	0.041	0.705	0.091	0.386
Xerostomia total score	0.089	0.398	0.007	0.949	0.026	0.809
Sticky saliva during day	0.260	0.010*	-0.104	0.339	0.006	0.959
Sticky saliva during night	0.244	0.016*	-0.038	0.729	0.007	0.947
Sticking saliva total score	0.301	0.003*	-0.093	0.392	0.008	0.943

*$p \leq 0.05$; M0: before treatment, M3: 3 months after treatment, M6: 6 months after treatment

Appendix 4. Spearman correlation coefficients of the WST versus the EORTC QLQ-H&N35

**WST**	***Number of swallows***						***Duration***					
**EORTC QLQ-H&N35**	***M0 (N=101)***		***M3 (N=92)***		***M6 (N=92)***		***M0 (N=101)***		***M3 (N=92)***		***M6 (N=92)***	
	Spearman's ρ	*p-value*	*Spearman's ρ*	*p-value*	*Spearman's ρ*	*p-value*	*Spearman's ρ*	*p-value*	*Spearman's ρ*	*p-value*	*Spearman's ρ*	*p-value*
Pain in mouth	0.088	0.378	0.175	0.094	-0.009	0.936	0.125	0.205	0.253	0.015*	-0.075	0.479
Swallowing	0.145	0.145	0.201	0.055	0.110	0.295	0.172	0.082	0.260	0.012*	0.176	0.094
Senses problems	0.056	0.575	0.055	0.601	-0.030	0.774	0.064	0.523	0.177	0.091	0.051	0.629
Speech problems	-0.043	0.667	0.173	0.099	0.110	0.295	0.070	0.482	0.291	0.005*	0.187	0.074
Trouble with social eating	0.126	0.206	0.113	0.285	0.279	0.007*	0.178	0.072	0.277	0.008*	0.302	0.003*
Trouble with social contact	0.050	0.615	0.135	0.201	0.156	0.138	0.104	0.297	0.274	0.008*	0.196	0.062
Less sexuality	0.152	0.128	-0.003	0.981	-0.017	0.877	0.166	0.096	-0.022	0.837	0.106	0.322
Teeth	-0.072	0.472	-0.013	0.903	0.024	0.819	0.015	0.883	0.085	0.421	0.098	0.359
Opening mouth	0.114	0.253	0.067	0.525	0.073	0.489	0.004	0.964	0.163	0.121	-0.047	0.656
Dry mouth	0.052	0.603	0.053	0.616	-0.085	0.422	0.013	0.897	0.177	0.091	-0.022	0.832
Sticky saliva	-0.121	0.227	0.107	0.308	-0.037	0.724	0.015	0.879	0.238	0.022*	0.077	0.467
Coughing	-0.018	0.859	0.035	0.741	-0.056	0.595	0.090	0.364	0.092	0.385	0.018	0.866
Feeling ill	0.082	0.413	0.169	0.108	0.161	0.127	0.029	0.775	0.282	0.007*	0.112	0.291
Pain killers	0.003	0.975	0.037	0.728	-0.109	0.303	0.047	0.637	0.020	0.847	-0.087	0.408
Nutritional supplements	0.052	0.607	0.086	0.417	0.156	0.137	0.093	0.348	0.192	0.067	0.151	0.150
Feeding tube	-0.106	0.289	-0.044	0.680	0.144	0.172	-0.073	0.461	0.030	0.775	0.117	0.266
Weight loss	-0.120	0.234	0.043	0.683	0.004	0.973	-0.024	0.810	0.053	0.618	0.031	0.773
Weight gain	-0.087	0.388	-0.170	0.108	0.108	0.306	-0.103	0.303	0.065	0.539	0.030	0.777

*:p≤0.05; M0: before treatment, M3: 3 months after treatment, M6: 6 months after treatment

Appendix 5. Spearman correlation coefficients of the WST versus the SWAL-QoL-NL

**WST**	***Number of swallows***						***Duration***					
**SWAL-QoL-NL**	***M0 (N=101)***		***M3 (N=92)***		***M6 (N=92)***		***M0 (N=101)***		***M3 (N=92)***		***M6 (N=92)***	
	Spearman's ρ	*p-value*	*Spearman's ρ*	*p-value*	*Spearman's ρ*	*p-value*	*Spearman's ρ*	*p-value*	*Spearman's ρ*	*p-value*	*Spearman's ρ*	*p-value*
General burden	0.175	0.084	0.156	0.135	0.083	0.430	0.026	0.806	0.205	0.049*	0.162	0.123
Food selection	0.106	0.292	0.226	0.029*	0.244	0.020*	0.110	0.299	0.332	0.001*	0.265	0.011*
Eating duration	0.172	0.087	0.204	0.051	0.336	0.001*	0.222	0.034*	0.315	0.002*	0.364	<0.001*
Eating desire	0.024	0.814	0.140	0.183	0.282	0.006*	0.171	0.104	0.322	0.002*	0.338	0.001*
Fear of eating	0.222	0.026*	0.217	0.038*	0.281	0.007*	0.210	0.044*	0.316	0.002*	0.399	<0.001*
Mental health	0.123	0.226	0.196	0.061	0.242	0.021*	0.143	0.175	0.211	0.044*	0.296	0.004*
Social functioning	0.159	0.118	0.214	0.041*	0.231	0.027*	0.106	0.314	0.280	0.007*	0.254	0.014*
Symptoms	0.248	0.013*	0.172	0.101	0.142	0.179	0.138	0.193	0.268	0.010*	0.255	0.015*
Total score	0.201	0.046*	0.238	0.023*	0.335	0.001*	0.194	0.064	0.353	0.001*	0.398	<0.001*

*:p≤0.05; M0: before treatment, M3: 3 months after treatment, M6: 6 months after treatment

Appendix 6. Spearman correlation coefficients of the WST versus the GRIX

**WST**	***Number of swallows***						***Duration***					
**GRIX**	***M0 (N=97)***		***M3 (N=85)***		***M6 (N=87)***		***M0 (N=97)***		***M3 (N=85)***		***M6 (N=87)***	
	Spearman's ρ	*p-value*	*Spearman's* ρ	*p-value*	*Spearman's* ρ	*p-value*	*Spearman's* ρ	*p-value*	*Spearman's* ρ	*p-value*	*Spearman's* ρ	*p-value*
Xerostomia during day	0.081	0.428	-0.071	0.513	-0.056	0.605	0.103	0.307	0.060	0.584	0.022	0.843
Xerostomia during night	0.115	0.257	0.095	0.374	-0.036	0.734	0.190	0.058	0.155	0.145	0.042	0.691
Xerostomia total score	0.098	0.341	0.025	0.820	-0.077	0.476	0.137	0.180	0.124	0.257	0.027	0.805
Sticky saliva during day	0.082	0.420	0.059	0.584	-0.048	0.652	0.185	0.064	0.149	0.161	0.080	0.453
Sticky saliva during night	0.103	0.307	-0.101	0.342	-0.066	0.529	0.244	0.014*	0.046	0.665	-0.001	0.990
Sticking saliva total score	0.075	0.459	-0.009	0.931	-0.107	0.315	0.212	0.034*	0.109	0.306	0.008	0.941

*:p≤0.05; M0: before treatment, M3: 3 months after treatment, M6: 6 months after treatment

Appendix 7. Spearman correlation coefficients of salivary flow versus the EORTC QLQ-H&N35

**Total salivary flow EORTC QLQ-H&N35**	***M0 (N=45)***		***M3 (N=68)***		***M6 (N=59)***	
	Spearman's ρ	*p-value*	*Spearman's ρ*	*p-value*	*Spearman's ρ*	*p-value*
Pain in mouth	-0.100	0.503	-0.171	0.153	-0.048	0.718
Swallowing	-0.050	0.737	-0.167	0.164	0.073	0.579
Senses problems	-0.450	0.002*	-0.272	0.022*	-0.287	0.026*
Speech problems	0.037	0.803	0.057	0.635	0.168	0.199
Trouble with social eating	-0.051	0.735	-0.292	0.014*	-0.238	0.067
Trouble with social contact	-0.179	0.228	-0.102	0.397	-0.008	0.950
Less sexuality	-0.024	0.875	-0.008	0.950	-0.181	0.171
Teeth	0.042	0.778	-0.034	0.775	-0.091	0.493
Opening mouth	-0.188	0.207	-0.174	0.146	0.093	0.479
Dry mouth	-0.227	0.126	-0.339	0.004*	-0.279	0.031*
Sticky saliva	-0.217	0.143	-0.321	0.006*	-0.350	0.006*
Coughing	-0.300	0.040*	-0.022	0.853	-0.074	0.572
Feeling ill	-0.125	0.403	0.024	0.843	0.124	0.351
Pain killers	0.011	0.940	0.056	0.645	-0.133	0.310
Nutritional supplements	-0.390	0.007*	-0.314	0.008*	-0.162	0.215
Feeding tube	-0.196	0.187	-0.189	0.114	NA	NA
Weight loss	-0.038	0.805	-0.105	0.384	-0.267	0.041*
Weight gain	-0.185	0.219	0.059	0.627	-0.127	0.335

*:p≤0.05; M0: before treatment, M3: 3 months after treatment, M6: 6 months after treatment

Appendix 8. Spearman correlation coefficients of salivary flow versus the SWAL-QoL-NL

**Total salivary flow SWAL-QoL-NL**	***M0 (N=45)***		***M3 (N=71)***		***M6 (N=57)***	
	Spearman's ρ	*p-value*	*Spearman's ρ*	*p-value*	*Spearman's ρ*	*p-value*
General burden	-0.145	0.347	-0.146	0.220	-0.032	0.810
Food selection	-0.147	0.337	-0.219	0.065	-0.312	0.015*
Eating duration	-0.218	0.150	-0.337	0.004*	-0.122	0.354
Eating desire	-0.182	0.231	-0.393	0.001*	-0.202	0.121
Fear of eating	-0.239	0.114	-0.148	0.218	-0.146	0.266
Mental health	-0.126	0.414	-0.273	0.021*	-0.235	0.070
Social functioning	-0.148	0.342	-0.322	0.006*	-0.224	0.086
Symptoms	-0.155	0.314	-0.194	0.105	-0.157	0.231
**Total score**	-0.218	0.156	-0.338	0.004*	-0.245	0.059

*:p≤0.05; M0: before treatment, M3: 3 months after treatment, M6: 6 months after treatment

Appendix 9. Spearman correlation coefficients of salivary flow versus the GRIX

**Total salivary flow**<br>**GRIX**	***M0***<br>***(N=45)***		***M3***<br>***(N=65)***		***M6***<br>***(N=57)***	
Xerostomia during day	-0.254	0.092	-0.332	0.006*	-0.441	0.001*
Xerostomia during night	-0.147	0.329	-0.225	0.063	-0.099	0.450
Xerostomia total score	-0.182	0.232	-0.327	0.008*	-0.320	0.015*
Sticky saliva during day	-0.218	0.151	-0.282	0.019*	-0.348	0.006*
Sticky saliva during night	-0.299	0.044*	-0.192	0.115	-0.128	0.331
Sticking saliva total score	-0.271	0.072	-0.274	0.023*	-0.256	0.048*

*:p≤0.05; M0: before treatment, M3: 3 months after treatment, M6: 6 months after treatment
